# Increased alternate splicing of *Htr2c* in a mouse model for Prader-Willi syndrome leads disruption of 5HT_2C_ receptor mediated appetite

**DOI:** 10.1186/s13041-016-0277-4

**Published:** 2016-12-08

**Authors:** Alastair S. Garfield, Jennifer R. Davies, Luke K. Burke, Hannah V. Furby, Lawrence S. Wilkinson, Lora K. Heisler, Anthony R. Isles

**Affiliations:** 1 Centre for Integrative Physiology, University of Edinburgh, Edinburgh, UK; 2 Behavioural Genetics Group, MRC Centre for Neuropsychiatric Genetics and Genomics, Neuroscience and Mental Health Research Institute, Schools of Medicine and Pscyhology, Cardiff University, Cardiff, UK; 3 Department of Pharmacology, University of Cambridge, Cambridge, UK; 4 Rowett Institute of Nutrition and Health, University of Aberdeen, Aberdeen, UK; 5 Present address: Cardiovascular and Metabolic Disease, Pfizer, Cambridge, MA 02139 USA

**Keywords:** Snord115, Prader-Willi syndrome, Serotonin 2C recptor, Alternate splicing, Feeding

## Abstract

Alternate splicing of serotonin (5-hydroxytryptamine; 5-HT) 2C receptor (5-HT_2C_R) pre-RNA is negatively regulated by the small nucleolar RNA, *Snord115*, loss of which is observed in nearly all individuals with Prader-Willi Syndrome (PWS), a multigenic disorder characterised by hyperphagia and obesity. Given the role of the 5-HT_2C_R in the regulation of ingestive behaviour we investigated the pathophysiological implications of *Snord115* deficiency on 5-HT_2C_R regulated appetite in a genotypically relevant PWS mouse model (PWS-IC). Specifically, we demonstrate that loss of *Snord115* expression is associated with increased levels of hypothalamic truncated 5-HT_2C_R pre-mRNA. The 5-HT_2C_R promotes appetite suppression via engagement of the central melanocortin system. *Pro-opiomelancortin* (*Pomc*) mRNA levels within the arcuate nucleus of the hypothalamus (ARC) were reduced in PWS-IC mice. We then went on to assess the functional consequences of these molecular changes, demonstrating that PWS-IC mice are unresponsive to an anorectic doses of a 5-HT_2C_R agonist and that this is associated with attenuated activation of POMC neurons within the ARC. These data provide new insight into the significance of *Htr2c* pre-mRNA processing to the physiological regulation of appetite and potentially the pathological manifestation of hyperphagia in PWS. Furthermore, these findings have translational relevance for individuals with PWS who may seek to control appetite with another 5-HT_2C_R agonist, the new obesity treatment lorcaserin.

**Electronic supplementary material:**

The online version of this article (doi:10.1186/s13041-016-0277-4) contains supplementary material, which is available to authorized users.

## Introduction

Manipulations of the central serotonin (5-hydroxytryptamine; 5-HT) system elicit profound effects on feeding behaviour [1]. Specifically, a reduction in serotonin availability or efficacy, causes hyperphagia and resultant weight gain, whilst augmented serotonin bioavailability or receptor-specific agonism leads to hypophagia and weight loss. Although the anorectic action of serotonin occurs via activation of multiple receptor subtypes, it is the 5-HT2C receptor (5-HT_2C_R) that is the most predominant [2]. Genetic ablation of the 5-HT_2C_R gene (*Htr2c*) in mice leads to hyperphagia [3], whilst receptor-specific agonists suppress food intake by enhancing the onset of satiety [4]. More recent investigation has identified the central melanocortin system as the principle mediator of 5-HT_2C_R regulated appetite [4–6]. The arcuate nucleus of the hypothalamus (ARC) contains two discrete populations of melanocortin neurons, those synthesising the anorectic melanocortin receptor (MCR) agonist pro-opiomelanocortin (POMC) and those synthesising the orexigenic MCR antagonist/inverse agonist agouti-related peptide (AgRP); 5-HT_2C_Rs are expressed on, and modulating firing of, ARC POMC neurons [7]. Furthermore, 5HT_2C_R expression specifically on POMC neurons is both necessary and sufficient for the promotion of satiety and the regulation of body weight [5, 8].

5-HT_2C_R function is influenced by two post-transcriptional processes, with the *Htr2c* pre-mRNA being subject to alternate splicing [9] and adenosine-to-inosine RNA-editing [10]. Both these events promote the translation of less functional receptors due to their effects on the amino acid sequence of the critical G-protein binding domain. Specifically, RNA-editing within exon V can result in the combinatorial conversion of five clustered adenosine residues into inosines, leading to a change in codon-specificity and subsequent amino acid sequence. Alternate splicing of exon V results in a truncated protein lacking a functional G-protein binding domain. However, although this truncated splice variant cannot act as a receptor it plays a critical role in overall 5-HT_2C_R function by forming a heterodimer and sequestering the full-length splice variant in the endoplasmic reticulum and reducing cell surface expression [11]. This role for the truncated 5-HT_2C_R has recently been confirmed and its functional importance demonstrated by microinjection into the brain of an oligonucleotide that promotes the production of the full-length transcript and, in turn, produces a change in feeding behaviour [12].

**Fig. 1:**
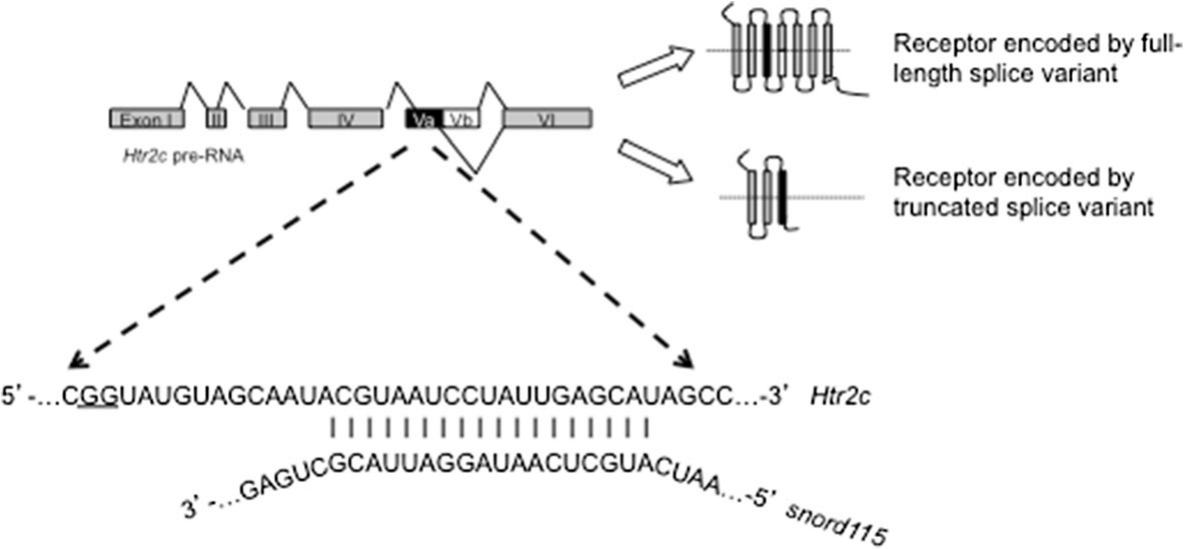
Schematic outlining the binding of *Snord115* to *Htr2c* and how alternate splicing can lead to full-length and truncated 5HT_2C_Rs. Binding of *Snord115* to a specific sequence in exon Va of the *Htr2c* pre-RNA promotes the inclusion of exon Vb and the production of the full-length 5HT_2C_R; the exon/alternative exon border in the proximal splice site (GG) is underlined. Skipping of exon Vb leads to the introduction of a premature “stop” codon and the production of a truncated 5HT_2C_R isoform. Loss of *snord115* expression, as is expected in the majority of cases of PWS, is expected to lead to an increase in levels of the truncated 5HT_2C_R isoform

Processing of *Htr2c* pre-RNA is mediated, in part, by the actions of the small nucleolar RNA (snoRNA) *Snord115* (previously *h/mbii-52*) [13, 14] present within the imprinted Prader-Willi syndrome (PWS) locus [15]. Loss of expression of the genes in this locus gives rise to PWS, a congenital neuroendocrine disorder in which hyperphagia and obesity are the hallmark symptoms [16]. A number of *in silico* and in vitro studies have demonstrated that this C/D box containing snoRNA primarily regulates alternate splicing [17], in particular the processing of *Htr2c* pre-mRNA, promoting the inclusion of exon Vb and reducing the amount of RNA encoding the truncated receptor (Fig. 1) [13]. Nevertheless, although increases in RNA-editing of the *HTR2C* pre-RNA has been shown in both PWS [13] and PWS mouse model brain samples [18], to our knowledge there has never been a clear demonstration of changes in alternate splicing.

Given the importance of 5-HT_2C_R function to the regulation of food intake, it has been suggested that loss of *SNORD115* expression may influence 5-HT_2C_R regulated appetite and could thus contribute to hyperphagia in PWS [13]. In this work, we examine the proportion of full-length and truncated splice variants of *Htr2c* mRNA in the PWS-IC mouse. The PWS-IC mouse is a full genetic model for PWS in which all paternally expressed genes in the cluster are silenced and in which we have previously demonstrated hyperphagia and abnormal feeding behaviour [18, 19]. We go on to assess the pathophysiological consequences of increased levels of truncated variant *Htr2c* on hypothalamic 5-HT_2C_R function, establishing an important role in the modulation of serotonin regulated appetite, and identify serotonin-melanocortin axis dysfunction as a potential contributing factor to hyperphagia in the clinically salient PWS-IC mouse.

## Results

### Increased truncated Htr2c splice variant in hypothalamus of PWS-IC mice

**Fig. 2:**
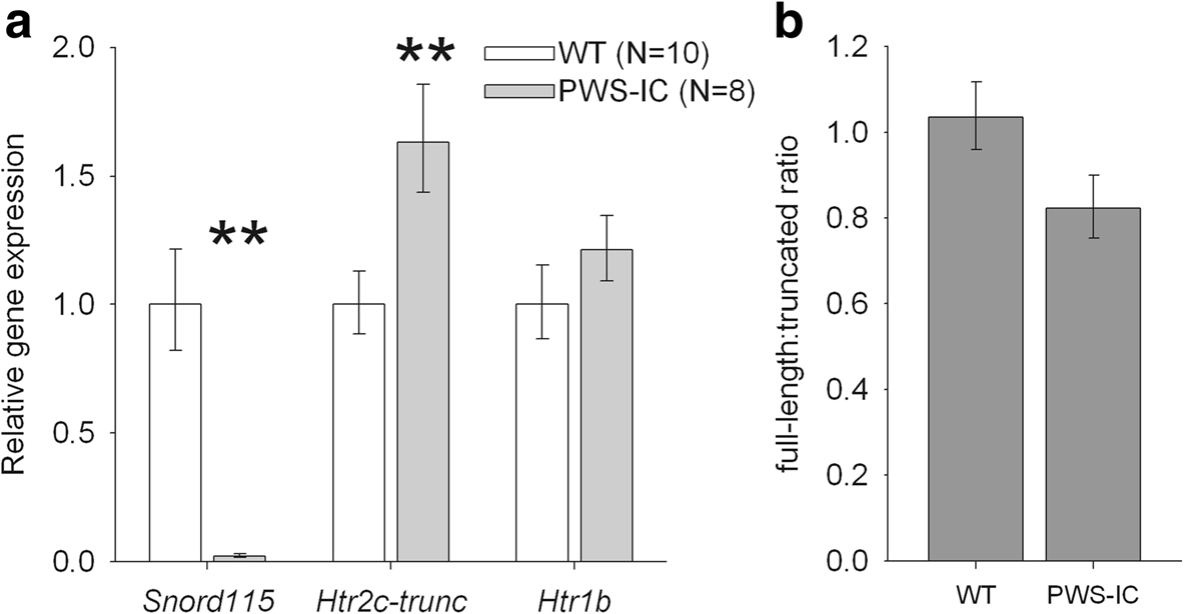
Increased levels of truncated *Htr2c* in PWS-IC hypothalamus. Gene expression and splice variant ratio was assessed using quantitative PCR (qPCR) in macro-dissected hypothalamus. **a** Expression of *Snord115* was absent in PWS-IC hypothalamus. This deficiency promotes alternate splicing of *Htr2c*, with levels of the truncated, non-functional, receptor isoform increased in PWS-IC mice relative to WT. The level of another regionally relevant serotonin receptor*, Htr1b* was unaltered in PWS-IC mice. **b** The consequence of increased levels of truncated *Htr2c* expression in PWS-IC hypothalamus, is a significant shift in the ratio of full-length:truncated, with a decreased proportion of functional (full-length) variants. Data presented as Mean ± SEM. Student’s *t*-test, *p < 0.05, **p < 0.01, **p < 0.001 compared to WT controls

Previous investigation of *Htr2c* pre-mRNA modification in PWS-IC mice at the whole brain level demonstrated an increase of RNA-editing in the absence of a significant effect on the presence of the truncated *Htr2c* mRNA isoform [18]. A more neuroanatomically refined analysis now reveals that virtually undetectable *Snord115* expression in the hypothalamus PWS-IC mice (Fig. 2a; t_16_ = 6.3, *p* = 0.001) is associated with a marked increase in levels of truncated *Htr2c* mRNA in the same samples (Fig. 2a; t_16_ = 3.1, *p* = 0.008). There was no significant difference in expression of 5-HT_1B_R (*Htr1b*) transcript (Fig. 2a; t_16_ = 0.8, *P* = 0.41). As expected, the increased levels of truncated *Htr2c* mRNA in turn resulted in a significant decrease in the ratio of full-length:truncated *Htr2c* in PWS-IC hypothalamus (Fig. 2b; one-tailed *t*-test, t_16_ = 2.17, *P* < 0.023).

### Altered hypothalamic neurochemistry in PWS-IC mice

**Fig. 3:**
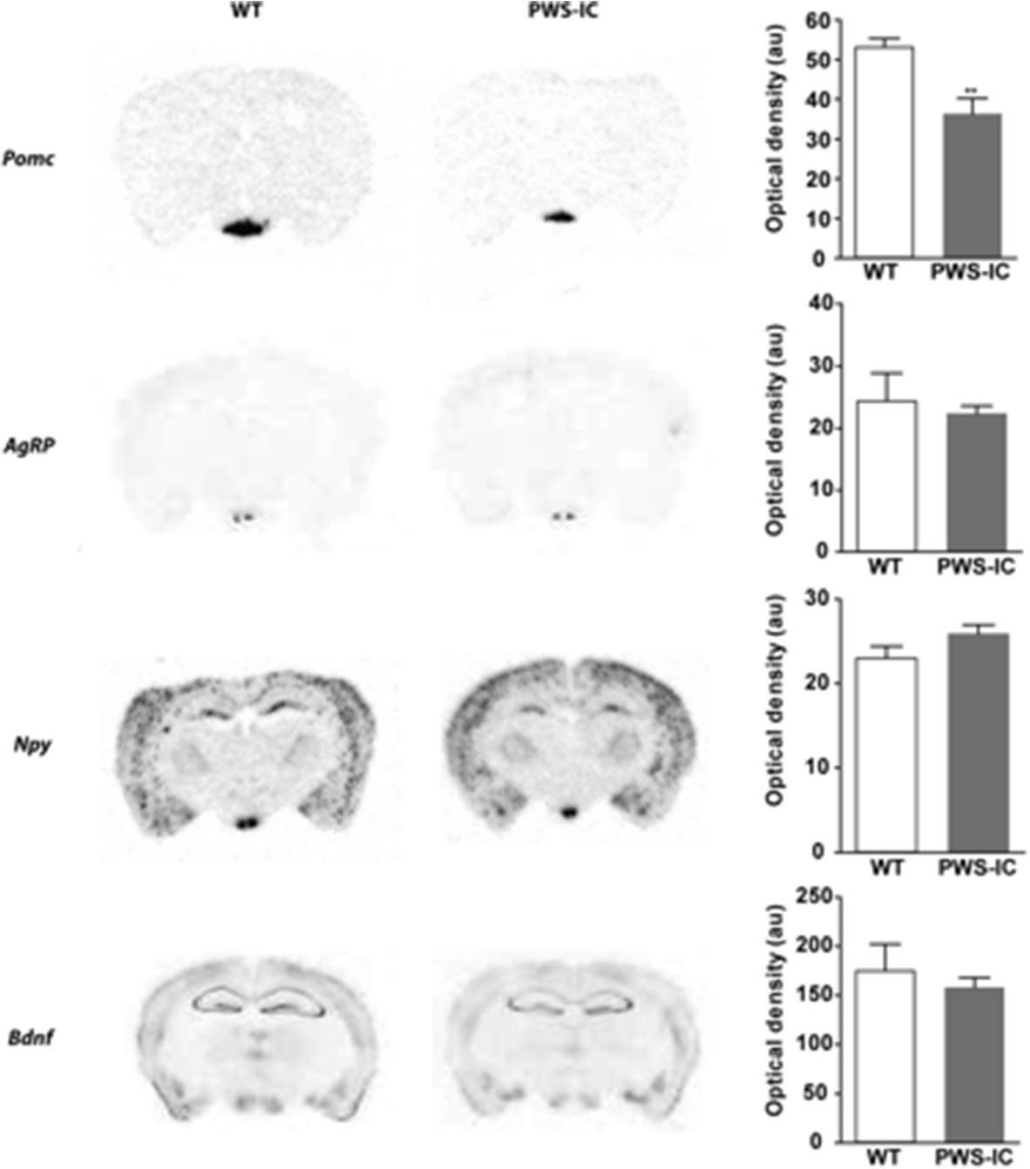
Perturbed hypothalamic neurochemistry in PWS-IC mice. In situ hybridisation analysis of ARC *Pomc*, *Agrp*, *Npy* and *Bdnf* mRNA expression in PWS-IC mice demonstrates a significant decrease in ARC *Pomc* mRNA expression, but no effect on ARC *Agrp*, *Npy* and *Bdnf* mRNA levels. Data presented as Mean ± SEM. Student’s *t*-test, *p < 0.05, **p < 0.01, **p < 0.001 compared to WT controls

In light of this, we undertook an assessment of hypothalamic neurochemistry in PWS-IC mice. Serotonin supresses appetite, a function mediated predominantly via 5-HT_2C_Rs of the ARC melanocortin system [2, 4–6]. Analysis of serotonin regulated appetite-related neuropeptide levels by in situ hybridisation histology (ISHH) and autoradiograph densitometry revealed a significant reduction in ARC *Pomc* mRNA expression (Fig. 3; t_6_ = 3.9, *p* = 0.008) but no change in that of agouti-related peptide (*Agrp*; t_6_ = 0.4, *p* = 0.681) or neuropeptides Y (*Npy,* t_4_ = 0.6, *p* = 0.14) mRNA expression in PWS-IC ARC, as compared to WT controls. Levels of brain-derived neurotrophic factor (*Bdnf*) mRNA within the ventromedial hypothalamus also did not vary between PWS-IC and WT mice (Fig. 3; t_4_ = 0.6, *p* = 0.58).

### Blunted anorectic effect of 5-HT_2C_R agonist in PWS-IC mice

**Fig. 4:**
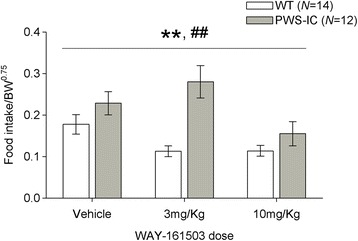
Blunted feeding effects of WAY-161503 in PWS-IC mice. 60 min food consumption upon administration of 3 mg/kg and 10 mg/kg WAY-161503 (s.c) in WT and PWS-IC mice. PWS-IC mice are unresponsive to an anorectic dose of the 5-HT_2C_R agonist WAY-161503. Data presented as Mean ± SEM, with statistical comparison performed by One-way repeated measure ANOVA, *p < 0.05 compared to vehicle controls

To directly assess the functional significance of increased levels of truncated *Htr2c* on 5-HT_2C_R regulated appetite we probed the response of PWS-IC mice to an anorectic dose of a 5-HT_2C_R specific agonist, WAY-161503 [20], in a post-fast re-feeding paradigm. Dosing with WAY-161503 produced a 20–25% decrease in food intake as expected (Fig. 4; repeated measures ANOVA, main effect of DOSE, F_2,48_ = 20.34, *p* = 0.001). As observed previously [19], PWS-IC mice consistently consumed more food than WT controls (Fig. 4; repeated measures ANOVA, main effect of GENOTYPE, F_1,24_ = 11.387, *p* = 0.003) and WT and PWS-IC mice responded differently to WAY-161503 (GENOTYPE X DOSE interaction, F_2,48_ = 5.68, *p* = 0.006). *Post hoc* (Tukey’s) analysis revealed that 3 mg/kg (*p* = 0.030) and 10 mg/kg (*p* = 0.033) WAY-161503 significantly reduced consumption in WT mice compared to vehicle treatment. However, no dose of WAY-161503 significantly reduced consumption relative to vehicle in PWS-IC mice (3 mg/Kg, *p* = 0.50; 10 mg/Kg, *p* = 0.26).

### 5-HT_2C_R agonist induced hypothalamic neuronal activation in PWS-IC mice

**Fig. 5:**
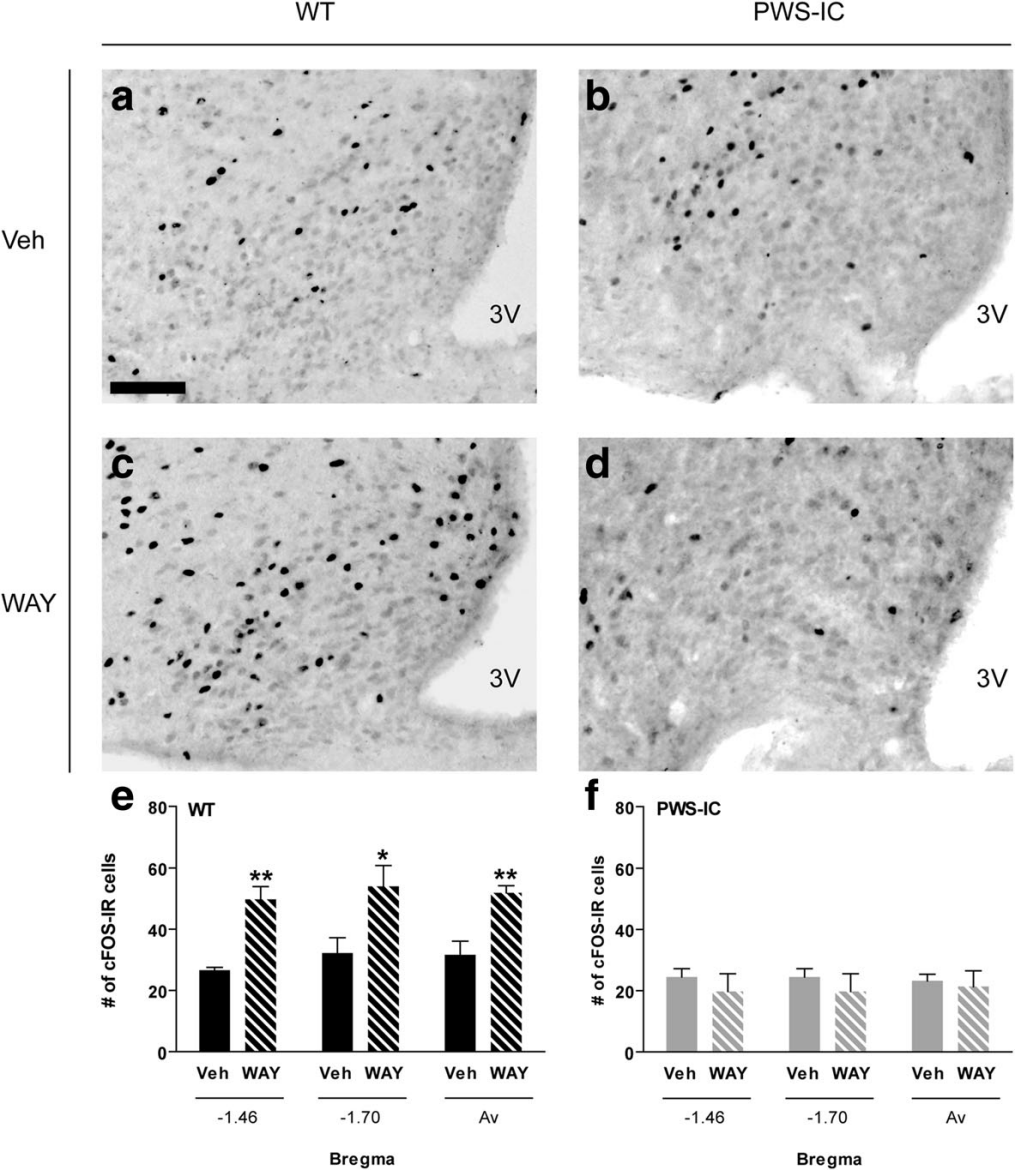
Decreased WAY-161503 induced cFOS induction in the ARC of PWS-IC mice. **a**−**d** Immunohistochemical detection of cFOS immunoreactivity (denoted by black nuclear staining) within the ARC (Bregma −1.46) following administration of (**a**, **b**) vehicle and (**c**, **d**) 3 mg/kg WAY-161503 to WT and PWS-IC mice. **e**, **f** Quantification of cFOS induction at two distinct neuroanatomical levels of ARC (−1.46 and −1.70 mm from bregma) and across the nucleus as a whole in (e) WT and (F) PWS-IC mice following WAY-161503 administration. PWS-IC mice exhibit attenuated ARC cFOS-IR upon WAY-161503 administration. Data presented as Mean ± SEM, with statistical comparison performed by Student’s *t*-test, *p < 0.05, **p < 0.01 compared to vehicle controls. Scale bar in (A), 100 μm and relates to all images. 3 V, third ventricle

**Fig. 6:**
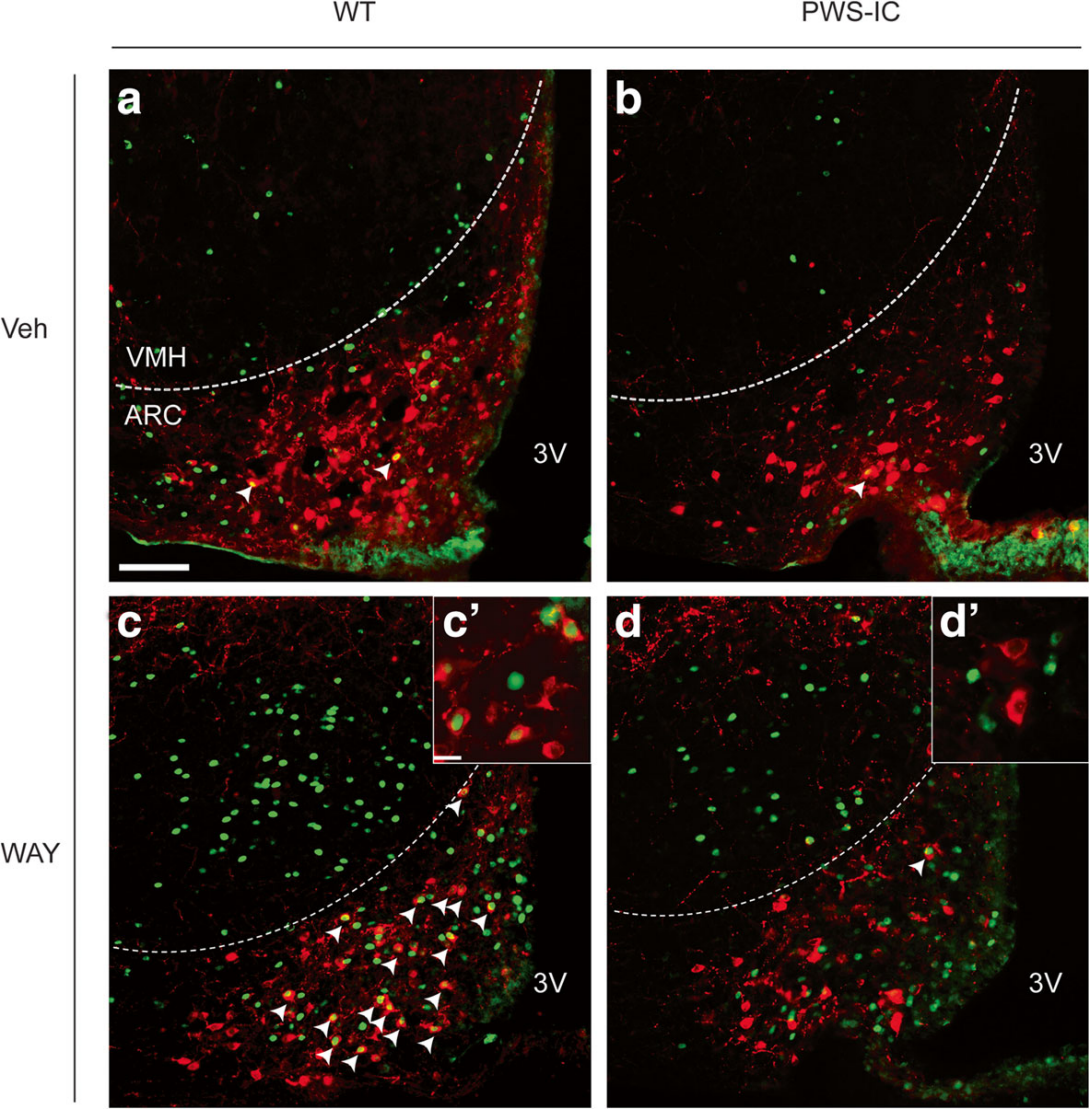
Decreased WAY-161503 induced activation of ARC POMC neurons in PWS-IC mice. Dual immunofluorescence histochemical colocalisation of cFOS (nuclear, green) and POMC (cytoplasmic, red) in the brains of (**a**, **c**) vehicle and (B, D) WAY-161503 treated (**a**, **c**) WT and (**b**, **d**) PWS-IC mice. Inserts in (**c**, **d**) represent high magnification images of colocalisation. WAY-161503 administration failed to activate ARC POMC neurons in PWS-IC mice. VMH, ventromedial nucleus of the hypothalamus; 3 V, third ventricle. Scale bar (a), 100 μm and relates to (**a**–**d**). Scale bar (C’), 25 μm and relates to (C’ and D’)

The appetite suppressing actions of 5-HT_2C_R agonists are fundamentally associated with the activation of neurons of the ARC [4, 5, 7]. As expected, 3 mg/kg WAY-161503 significantly increased the overall number of cFOS-immunoreactive (IR) cells within the ARC (Fig. 5; two-way ANOVA, main effect of DOSE, F_1,15_ = 7.13, *P* = 0.020). However, there was also an interaction between DOSE and GENOTYPE (two-way ANOVA, F_1,15_ = 9.76, *P* = 0.009). Analysis of cell counts in WT brain, at two separate neuroanatomical levels of the ARC (−1.46 and −1.70 mm from bregma), as well as across the rostral-caudal extent of the nucleus, revealed a robust and significant increase in cFOS induction (Fig. 5a, c, e; bregma −1.46, t_5_ = 5.3, *p* = 0.010; bregma −1.70, t_6_ = 2.6, *p* = 0.042; Av, t_6_ = 4.0, *p* = 0.007). In contrast, WAY-161503 administration failed to induce cFOS-IR at any level of the ARC in PWS-IC mice (Fig. 5b, d, f; bregma −1.46, t_6_ = 0.3, *p* = 0.75; bregma −1.70, t_6_ = 0.7, *p* = 0.487; Av, t_6_ = 0.8, *p* = 0.87). Further investigation revealed the reduced anorectic efficacy of WAY-161503 in PWS-IC mice to be associated with a failure to induce appropriate downstream POMC signalling as administration of 3 mg/kg WAY-161503 substantially increased the number of cFOS-IR POMC neurons within the ARC of WT (Fig. 6a, c, C’), but not PWS-IC mice (Fig. 6b, d, D’).

## Discussion

The contribution of the 5-HT_2C_R to the central regulation of energy balance, together with the established role of *Snord115* in modulating 5-HT_2C_R function and lack of *SNORD115* expression in nearly all cases of PWS raises the possibility that abnormalities in 5-HT_2C_R-mediated feeding circuitries in brain may contribute to the overeating seen in PWS subjects. Using a model for PWS that lacks *Snord115* expression, the PWS-IC mouse, we provide evidence for this idea, showing that the predicted increases in levels of truncated *Htr2c* in brain. Moreover, these molecular and behavioural effects are further associated with evidence of reduced function in specific brain circuits, influenced by 5-HT_2C_R, that control feeding.

Although a change in full-length:truncated *HTR2C* ratio as a consequence of *SNORD115* loss has been predicted for PWS [13], our previous investigation of *Htr2c* pre-mRNA modification in PWS-IC mice at the whole brain level demonstrated an increase of RNA-editing in the absence of a significant effect on proportion of splice variants [18]. However, more neuroanatomically refined analysis of the hypothalamus now reveals concomitant increased levels of the truncated *Htr2c* mRNA with loss of *Snord115* expression in the PWS-IC mice. The truncated variant of 5HT_2C_R can dimerize with the full-length receptor protein, sequestering it to the endoplasmic reticulum [11]. An increase in the abundance of the truncated variant, as occurs in the hypothalamus of PWS-IC mice, would therefore alter the functionality of the serotonin signalling via 5HT_2C_R.

To directly assess the contribution of reduced 5-HT_2C_R signalling on appetite behaviour in this model for PWS, PWS-IC mice were treated with an anorectic dose of a 5-HT_2C_R specific agonist [20]. Our finding that PWS-IC mice exhibited insensitivity to the anorectic effects of WAY-161503 was correlated with suppressed agonist-induced cFos-IR in ARC POMC neurons of PWS-IC mice. Although we anticipate altered reactivity to WAY-161503 throughout the brain of PWS-IC mice, we focused on ARC neurons because of the well-established link between 5HT_2C_R expression on POMC neurons and satiety and the regulation of body weight [5, 8]. When considered in the context of the basal feeding behaviour of PWS-IC mice [19], these data suggest that *Snord115* deficiency derives less functional 5-HT_2C_Rs that are incapable of effectuating the anorectic actions of serotonin (via stimulation of downstream POMC signalling) and highlights the independent contribution of this axis to the hyperphagic nature of PWS-IC mice.

A role for abnormal melanorcortin signalling in PWS was underlined by ISSH analysis that demonstrated a significant decrease in ARC *Pomc* mRNA, but no differential expression of *Agrp* or *Npy* in PWS-IC mice. This observation is supported by data from 5-HT_2C_R deficient mice [21] and 5-HT_2C_R-VGV knock-in mice that constitutively express the fully edited isoform of 5HT_2C_R [22], both of which exhibit a reduction in *Pomc* mRNA levels. Together these data suggest that perturbed melanocortinegric signalling may be a functional target for the PWS-IC mutation. Interestingly, elevated *Pomc* expression is observed in neonatal PWS-IC mice and has been implicated in the depressed feeding and early postnatal lethality observed of these mutants [23].

For some time there has been speculation with regards to the importance of 5HT_2C_R mediated appetite in PWS due to the loss of the snoRNA *SNORD115* which negatively regulates the generation of a truncated splice variant of this receptor [13]. To our knowledge this is the first in vivo demonstration that increased levels of truncated *Htr2c* in a mouse model for PWS promotes a disruption of 5-HT_2C_ receptor-mediated appetite. Although PWS symptoms observed in very rare cases with an intact *SNORD115* locus discounts this gene as causal of PWS *per se* [24, 25], our data suggest that 5HT_2C_R dysfunction may also contribute to hyperphagia in the majority of cases of PWS where *SNORD115* expression is lost. However, the PWS-IC model used here also lacks expression of all paternally expressed genes from the PWS imprinting cluster [26]. Consequently, we cannot exclude the involvement the loss of *Necdin* expression in causing alterations in the serotonergic system of the PWS-IC model, as specific knockout of this gene in mice also leads to altered serotonergic neurochemistry [27] although not, as far as we are aware, changes in *Htr2c* splicing. Nevertheless, the data presented here thereby provide new insight into the significance of *Htr2c* pre-mRNA processing to the physiological regulation of appetite and potentially the pathological manifestation of hyperphagia in PWS. Furthermore, these findings have translational relevance for individuals with PWS who may seek to control appetite with another 5-HT_2C_R agonist, namely the new obesity treatment lorcaserin [28].

## Methods

### Animals

PWS-IC animals [29] were bred by transmitting the imprinting centre (IC) mutation paternally (i.e. PWS-IC^+/−^). PWS-IC and WT littermates were housed in single-sex groups, were subject to a 12 h light/dark cycle, and had *ad libitum* access to standard chow and water, unless stated otherwise.

### Feeding studies

Daily consumption of a standard (3.68 kcal/g –fat 10%, protein 20%, carbohydrate 70%) and high-fat diet (4.54 kcal/g –fat 45%, protein 20%; carbohydrate 35% - Special Diet Services, Witham, Essex, UK) was assessed over two consecutive 7-day periods. Food consumption was normalised to body weight^0.75^ (BW^0.75^).

### Analysis of Htr2c mRNA processing

Whole hypothalamus was macro-dissected using a protocol established in the laboratory [30] and RNA isolated using standard Trizol methods and reverse-transcribed using SuperScript® III First-Strand Synthesis SuperMix (Invitrogen). Gene expression was assessed using qPCR, performed with custom designed primers and was analysed using the ΔCt method, normalising to 18 s rRNA as described previously [18]. Ratios of full-length:truncated *Htr2c* were calculated using a modified ΔCt method as follows:

$$ Htr2c\hbox{-} \mathrm{full}-Htr2c\hbox{-} \mathrm{trunc} $$

All data were then transformed as usual (2^-Δ*C*T^) before statistical analysis. WT *n* = 10, PWS-IC *n* = 8.

### In situ hybridisation histochemistry (ISHH)

Adult brain tissue was collected from animals transcardially perfused with 10% formalin, cryoprotected in 20% sucrose and sectioned at 25 μm on a freezing microtome. Tissue was processed for in situ hybridisation as previously described [31]. Radiolabelled riboprobes specific to the mRNA sequences of *Pomc*, *Agrp*, *Npy* and *Bdnf* were used to detect gene expression [7, 32, 33]. Riboprobes were synthesized by PCR using cDNA obtained from normal mouse brain. Recombinant plasmids were linearised by restriction digest and subjected to in vitro transcription with a T7 RNA polymerase in the presence of ^35^S-labeled UTP, according to the manufacturer’s instructions (Ambion Inc, Austin, Tx). Riboprobes were diluted to 2x10^7^ cpm/ml in hybridization solution. Before hybridization, brain sections were fixed in 4% formaldehyde in DEPC-treated PBS and permeabilised by heating in prewarmed sodium citrate buffer (95–100 °C, pH 6.0), before being dehydrated in ascending concentrations of ethanol, and air-dried. Probe solution was applied to each slide and sections were incubated for 12–16 h at 57 °C. After this time slides were, incubated in 0.002% RNase A (Qiagen, Valencia, CA), followed by sequential washes in decreasing concentrations of sodium citrate buffer (SSC). The sections were dehydrated in ascending concentrations of ethanol with 0.3 M ammonium acetate (NH_4_OAc) followed by 100% ethanol. Slides were air-dried and placed in X-ray film cassettes with BMR-2 film (Kodak, UK) for 72 h and developed on an OPTIMAX X-ray film processor. For assessment of gene expression in the autoradiograph films were subjected to densitometry analysis using ImageJ. Integrated mean density values were calculated for each section containing complete ARC.

For *Pomc* and *Npy* analysis, *n* = 4 for both WT and PWS-IC groups. For *AgRp* and *Bdnf* analysis *n* = 3 for both groups.

### WAY-161503 induced cessation of feeding

WAY-161503 hydrochloride (Tocris, Missouri USA) was dissolved in distilled water. The study was conducted as a within-subjects Latin square design, with at least 72 h between treatments. Food was removed at the onset of the dark cycle and the animals fasted for 16 h (water was available *ad libitum*). The following morning male and female WT (*n* = 14) and PWS-IC (*n* = 12) animals were treated with a single injection (sub cutaneous, s.c.) of either vehicle or WAY-161503 (3 and 10 mg/kg) and 15 min later presented with a known weight of mash and allowed to freely consume for 60 min. At the end of the test the remaining food (plus any spillage) was re-weighed. Food consumption was normalised to body weight^0.75^ (BW^0.75^).

### WAY-161503 induced cFOS immunoreactivity (cFOS-IR)

Animals were treated (s.c.) with either 3 mg/kg WAY-161503 or vehicle and brains collected 2 h later after transcardial perfusion with 10% formalin. Brains were cryoprotected in 20% sucrose and sectioned at 25 μm on a freezing microtome. Free-floating sections were treated as previously described [4, 7]. Briefly, sections were incubated overnight at room temperature in blocking solution containing rabbit anti-cFOS antibody (Calbiochem; 1:8000), washed, and transferred to blocking solution containing biotinylated donkey anti-rabbit secondary antibody. After rinsing, staining was visualised using following 3,3′-Diaminobenzidine (DAB). For quantification of cFOS-IR, sections containing ARC were assigned a bregma level and the boundaries of the ARC delineated based on neuro-architecture and the Mouse Brain Atlas [34]. ARC cFOS-IR cells falling within the defined regions were counted unilaterally and a bilateral average for the nucleus calculated. For all groups (WT saline, PWS-IC saline, WT WAY-162503, PWS-IC WAY-162503) *n* = 4.

### Dual Immunofluorescence Histochemistry (IHC)

For co-localisation of cFOS-IR and POMC-IR, sections were first treated as detailed above for cFOS-IR and then processed for POMC immunofluorescence (rabbit anti-POMC – Immunostar, 1:1000; anti-rabbit Alexa Fluor-568 – Molecular Probes, 1:1000). For cFOS/POMC colocalisation sections were imaged on a Zeiss Axioskop 2 microscope and a Zeiss AxioCam HRc digital camera. Chromogenic cFOS signals were photographed in brightfield and fluorescent POMC signal under appropriate excitation wavelength and the images merged in Photoshop CS3 (Adobe Inc).

### Statistics

All data were analysed using SPSS 20 (SPSS, USA). Data were analysed by Student’s *t*-test (two-way, unless otherwise indicated) or mixed ANOVA, with between subjects factor of GENOTYPE (PWS-IC vs. WT), and within subject factor DOSE (vehicle, 3 mg/kg and 10 mg/kg WAY-161503). All significance tests were performed at alpha level of 0.05 and where significant interactions were identified in the main ANOVA, *post-hoc* tests using appropriate pair-wise comparisons were performed. For repeated measures analyses, Mauchly’s test of sphericity of the covariance matrix was applied.

## Conclusion

Alternate splicing of the serotonin 2C receptor (5-HT_2C_R) is negatively regulated by the *Snord115*, loss of which is seen in most patients with Prader-Willi syndrome (PWS). Given the role of serotonin in the regulation of ingestive behaviour we investigated the pathophysiological implications of *Snord115* deficiency showing that increased levels of a truncated isoform of 5-HT_2C_R in the hypothalamus leads to abnormal 5-HT_2C_R-mediated appetite in a mouse model for PWS. We conclude that loss of *Snord115* expression is physiologically relevant to 5-HT_2C_R mediated appetite which in turn contributes to general hyperphagia in most cases of PWS. These findings also are important for individuals with PWS who may seek to control appetite with the new obesity treatment lorcaserin.

## Additional files

Additional file 1: Hypothalamus qPCR analysis. (XLSX 28 kb)
Additional file 2: Pomc, AgRP, Npy, Bdnf densitometry. (XLSX 11 kb)
Additional file 3: PWS feeding and serotonin pharmacology. (XLS 173 kb)
Additional file 4: WAY 0 v 3mg/kg ARC cFos. (XLSX 17 kb)

## Abbreviations

5HT_2C_R: 5-hydroxy-tryptamine 2C receptor
ANOVA: Analysis of variance
ARC: Arcuate nucleus of the hypothalamus
BW: Body weight
DAB: 3,3′-Diaminobenzidine
IHC: Immunohistochemistry
ISSH: In situ hybridisation histology
MCR: Melanocortin receptor
POMC: Pro-opiomelanocortin
PWS: Prader-Willi Syndrome
WT: Wild-type
